# Can initial chest CT scan predict status and clinical outcomes of COVID-19 infection? A retrospective cohort study

**DOI:** 10.1186/s43055-021-00538-6

**Published:** 2021-06-28

**Authors:** Iman Abdollahi, Mehrdad Nabahati, Mostafa Javanian, Hoda Shirafkan, Rahele Mehraeen

**Affiliations:** 1 Student Research Committee, Babol University of Medical Sciences, Babol, Iran; 2 Department of Radiology, Shahid Beheshti Hospital, Babol University of Medical Sciences, Ganjafrooz Street, Babol, Mazandaran Iran; 3 Infectious Diseases and Tropical Medicine Research Center, Health Research Institute, Babol University of Medical Sciences, Babol, Iran; 4 Social Determinants of Health Research Center, Health Research Institute, Babol University of Medical Sciences, Babol, Iran

**Keywords:** Computed tomography, Coronavirus disease 2019, Clinical outcome

## Abstract

**Background:**

We aimed to investigate the association of initial chest CT scan findings with status and adverse outcomes of COVID-19 (including ICU admission, mortality, and disease severity).

This retrospective cohort study was performed in three hospitals in Babol, northern Iran, between February and March 2020. Cases were confirmed by real-time polymerase chain reaction (RT-PCR). Clinical and paraclinical data of the patients were collected from their medical records. CT severity score (CSS) was calculated by a senior radiologist. Disease severity was determined based on the World Health Organization criteria.

**Results:**

In total, 742 patients were included, of whom 451 (60.8%) were males and 291 (39.2%) were females. The mean age was 56.59 ± 14.88 years old. Also, 523 (70.5%) were RT-PCR-positive. Ground glass opacity was directly associated with RT-PCR positivity (odds ratio [OR] = 2.07). Also, RT-PCR-positive cases had significantly a higher CSS than RT-PCR-negative cases (*p* = 0.037). In patients confirmed with COVID-19, peribronchovascular distribution of lesions, number of zones involved, and CSS were associated with increased risk of ICU admission (OR = 2.93, OR = 2.10, and OR = 1.14, respectively), mortality (OR = 2.30, OR = 1.35, and OR=1.08, respectively), severe disease (OR = 2.06, OR = 1.68, and OR = 1.10, respectively), and critical disease (OR = 4.62, OR = 3.21, and OR = 1.23, respectively). Also, patients who had consolidation were at a higher risk of severe disease compared with those who did not (OR = 4.94).

**Conclusion:**

Initial chest CT scan can predict COVID-19 positivity, ICU admission, mortality, and disease severity, specifically through CSS.

## Background

Coronavirus disease 2019 (COVID-19), which is caused by the novel severe acute respiratory syndrome coronavirus 2 (SARS-CoV-2) [1], has already infected more than 146 million people and killed more than 3 million people around the world [2], and these rates are increasing.

According to the guidelines, the gold standard for diagnosis of COVID-19 is real-time polymerase chain reaction (RT-PCR) testing on specimens obtained from respiratory tract [3]. However, the accuracy of this method is debatable due to false positive and negative results observed in different settings [4]. Also, the relatively slow process in providing the results makes RT-PCR less ideal in clinical practices [3, 4]. Therefore, accompanying techniques for earlier diagnosis of the patients are needed. Chest computed tomography (CT) scan is an important method for the diagnosis of COVID-19 pneumonia [5]. Being more time-saving and having a comparable sensitivity versus RT-PCR assay make CT scan a powerful tool for rapid screening of the suspected cases [6, 7].

A number of studies have stated that findings of chest CT scan are potentially predictive for the clinical outcomes of the patients, suggesting CT scan as a helpful tool in diagnosis and management of the patients with COVID-19 [8, 9]. However, a notable limitation of those studies is a small sample size, potentially leading to decreased reliability of evidence. Also, to our knowledge, the number of studies that used the scoring system to quantify lung involvement is limited. Moreover, not enough information exists on the predictive ability of CT scan results for status of COVID-19 infection. To overcome these issues, we aimed to conduct a study to investigate the association of CT scan findings with status and adverse outcomes of COVID-19 (including ICU admission, mortality, and disease severity).

## Methods

### Locations and patients

This retrospective cohort study was performed in three hospitals affiliated to Babol University of Medical Sciences, including Rohani, Shahid Beheshti, and Yahyanejad hospitals, between February and March 2020. Babol was one of the first cities in north of Iran with confirmed cases. We initially included the individuals presenting with suspicious symptoms (fever, respiratory symptoms, such as cough, dyspnea, and sputum) who were referred to the emergency unit and underwent necessary clinical and paraclinical assessments for COVID-19. Chest CT scan was performed on all suspected individuals after triage. For RT-PCR testing, trained technicians collected nasopharyngeal swab specimens and sent them to the laboratories of Babol health center. RT-PCR was done for all triaged patients. The time gap between the nasopharyngeal swab sampling and CT scan was less than 12 h.

Patients with chest CT scans suggestive of COVID-19 and at least one of the following criteria were admitted to the hospitals, according to the national COVID-19 protocol: hypoxia (O2 saturation ≤ 92%), tachypnea (respiratory rate ≥ 22), tachycardia (pulse rate > 100), or hypotension (systolic blood pressure 100 mmHg or less). The following data were extracted from the patients’ medical records by a trained research team: demographic information (such as sex and age), comorbidities (such as cardiovascular diseases [CVDs], asthma, chronic obstructive pulmonary disease [COPD], and diabetes). Cases with incomplete information about comorbidities and/or RT-PCR results, as well as those declined to participate in the study, were excluded from further investigations. We categorized clinically the COVID-19 pneumonia into different disease severities (moderate, severe, critical) based on the definition by World Health Organization (WHO) [10]. Patients with mild disease were not admitted as per the national COVID-19 protocol.

### Imaging collection and analysis

The patients underwent non-enhanced 16-detector-row CT scan during deep inspiration breath-hold in the supine position (Siemens SOMATOM Emotion 16, Siemens Healthcare, Med Image Systems, Germany). The scanning parameters were as follows: tube voltage, 100 KV for patients with BMI ≤ 30 and 120 KV for patients with BMI > 30; tube current, 50–100 mAs; pitch, 0.8–1.5; thickness, 1–3mm; Matrix, 512. Additional image reconstructions were not necessary. The CT scans were evaluated by a single radiologist with an experience of more than 15 years (R.M.), who was blinded to the patients’ status. The following imaging characteristics were recorded: ground glass opacity, consolidation, reticular pattern, lesions distribution (peribronchovascular or peripheral), side of lung involvement, crazy paving, pleural effusion, number of lung zones involved, cavity, and tree-in-bud pattern.

The CT severity score (CSS) for each patient was calculated based on the percentage of lung zones involvement [11]. In this regard, right and left lungs were divided into three (upper, middle, and lower) and two (upper and lower) zones, respectively. The scoring system was as follows: score 0 representing no involvement, score 1 representing < 5% involvement, score 2 representing 5–25% involvement, score 3 representing 26–50% involvement, score 4 representing 51–75% involvement, score 5 representing > 75% involvement. Finally, sum of the scores yielded the total CSS, ranging from 0 to 25.

### Data analysis

The statistical analyses were performed by SPSS software. The obtained data initially underwent descriptive analyses. To assess normality of the data, Kolmogorov-Smirnov test was used. For comparing parametric and non-parametric continuous data between the groups, independent t test and Mann–Whitney test were used, respectively. We performed chi-squared test and logistic regression analysis to investigate the association of baseline information and imaging findings of the patients with study outcomes (COVID-19 status and adverse disease outcomes). The factors with significant association were entered into the multivariable analysis. The results were presented as odds ratio (OR) as well as 95% confidence interval (CI). We also calculated the area under the curve (AUC) to estimate the predictive ability of CT scan features for the study outcomes. A *p* value less than 0.05 was considered statistically significant.

## Results

### Basic information

Initially, 829 patients were admitted to the hospitals, of whom 87 cases were excluded from the study due to meeting exclusion criteria. Totally, 742 patients were included for further assessments, of whom 451 (60.8%) were males and 291 (39.2%) were females. The mean age was 56.59 ± 14.88 years old, ranging from 23 to 93 years old. The patients’ symptoms included fever (68.2%), chills (60.7%), myalgia (46.3%), headache (22.5%), dry cough (57.6%), sputum (21.9%), sore throat (15.2%), and nasal congestion (5.1%). Cardiovascular diseases were the most prevalent comorbidity observed in the patients (*n* = 219, 41.9%). Regarding COVID-19 status, 523 (70.5%) were RT-PCR-positive and others were RT-PCR-negative. Of 523 patients confirmed with COVID-19, 163 (31.2%) were admitted to the ICU and 360 (68.8%) were admitted to the regular ward. Also, 402 cases (76.7%) survived and were discharged, while 121 patients (23.3%) died. In terms of disease severity, 242 patients (46.3%) had moderate disease, 155 patients (29.6%) had severe disease, and 126 patients (24.1%) had critical disease.

### Imaging findings and COVID-19 status

Table 1 shows distribution of the baseline information of the patients by COVID-19 status. Also, the findings of chest CT scan according to COVID-19 status are represented in Table 2. Due to the small number of cavity (*n* = 4) and tree-in-bud pattern (*n* = 3) found in CT scan, they were excluded from further analyses. Analyses demonstrated that ground glass and consolidation were significantly higher in COVID-19-positive patients than in COVID-19 negative patients (OR = 2.92 and OR = 2.40, respectively). After adjustment for imaging findings, ground glass was directly associated with COVID-19 infection (OR =2.07, AUC = 54.8%). The sensitivity, specificity, and accuracy of ground glass opacity for the infection were 91.1%, 15.5%, and 70.9%, respectively. It has also been found that RT-PCR-positive cases had significantly a higher CSS than RT-PCR-negative cases (OR = 1.03). The median number of CSS was 18 (interquartile range 12.75–22) and we used it as a threshold for the relevant analyses. Based on the analyses, CSS ≥ 18 could predict COVID-19 infection in the study cases (OR = 1.39, AUC = 54.1%). No significant associations were identified between COVID-19 infection and other CT findings, such as reticular pattern, bilateral lung involvement, crazy paving, pleural effusion, lesions distribution, and number of zones involved.

**Table 1:** Distribution of baseline information by real-time polymerase chain reaction (RT-PCR) results

Variables	RT-PCR-positive (***n*** = 523)	RT-PCR-negative (***n*** = 219)	***P*** value
**Age (years), mean ± SD**	56.55 ± 14.75	56.72 ± 15.23	0.885
**Sex, n (%)**			
**Male**	317 (60.6)	134 (61.2)	0.884
**Female**	206 (39.4)	85 (38.8)	
**CVDs, n (%)**			
**Yes**	219 (41.9)	93 (42.5)	0.882
**No**	304 (58.1)	126 (57.3)	
**Asthma/COPD, n (%)**			
**Yes**	52 (9.9)	19 (8.7)	0.593
**No**	471 (90.1)	200 (91.3)	
**Diabetes, n (%)**			
**Yes**	130 (24.9)	52 (23.7)	0.748
**No**	393 (75.1)	167 (76.3)	

*CVDs* cardiovascular diseases, *COPD* chronic obstructive pulmonary disease

**Table 2:** Association between imaging findings and real-time polymerase chain reaction (RT-PCR) results

Imaging findings	RT-PCR-positive (***n*** = 523)	RT-PCR-negative (***n*** = 219)	OR (95% CI)	***P*** value
**Ground glass, n (%)**				
No	31 (5.9)	34 (15.5)	1	
Yes	492 (94.1)	185 (84.5)	2.92 (1.74–4.88)	< 0.001
**Consolidation, n (%)**				
No	47 (9.0)	42 (19.2)	1	
Yes	476 (91.0)	177 (80.8)	2.40 (1.53–3.77)	< 0.001
**Reticular pattern, n (%)**				
No	394 (75.3)	171 (78.1)	1	
Yes	129 (24.7)	48 (21.9)	1.16 (0.80–1.70)	0.423
**Bilateral lung involvement, n (%)**				
No	26 (5.0)	10 (4.6)	1	
Yes	497 (95.0)	209 (95.4)	0.91 (0.43–1.93)	0.815
**Crazy paving, n (%)**				
No	412 (78.8)	170 (77.6)	1	
Yes	111 (21.2)	49 (22.4)	0.93 (0.64–1.36)	0.728
**Pleural effusion, n (%)**				
No	481 (92.0)	205 (93.6)	1	
Yes	42 (8.0)	14 (6.4)	1.27 (0.68–2.39)	0.441
**Lesions distribution, n (%)**				
Peribronchovascular				
No	348 (66.5)	148 (67.6)	1	
Yes	175 (33.5)	71 (32.4)	1.05 (0.75–1.47)	0.784
Peripheral				
No	19 (3.6)	6 (2.7)	1	
Yes	504 (96.4)	213 (97.3)	0.74 (0.29–1.89)	0.539
**Number of zones involved, median (IQR)**	5 (4–5)	4 (4–5)	1.03 (0.88–1.22)	0.717
**CSS (continuous), median (IQR)**	19 (13–23)	18 (12–21)	1.03 (1.01–1.06)	0.037
**CSS ≥ 18, n (%)**				
No	232 (44.4)	115 (52.5)	1	
Yes	291 (55.6)	104 (47.5)	1.39 (1.01–1.90)	0.042

*OR* odds ratio, *CI* confidence interval, *IQR* interquartile range

### Imaging findings and outcomes of COVID-19 patients

**Fig. 1:**
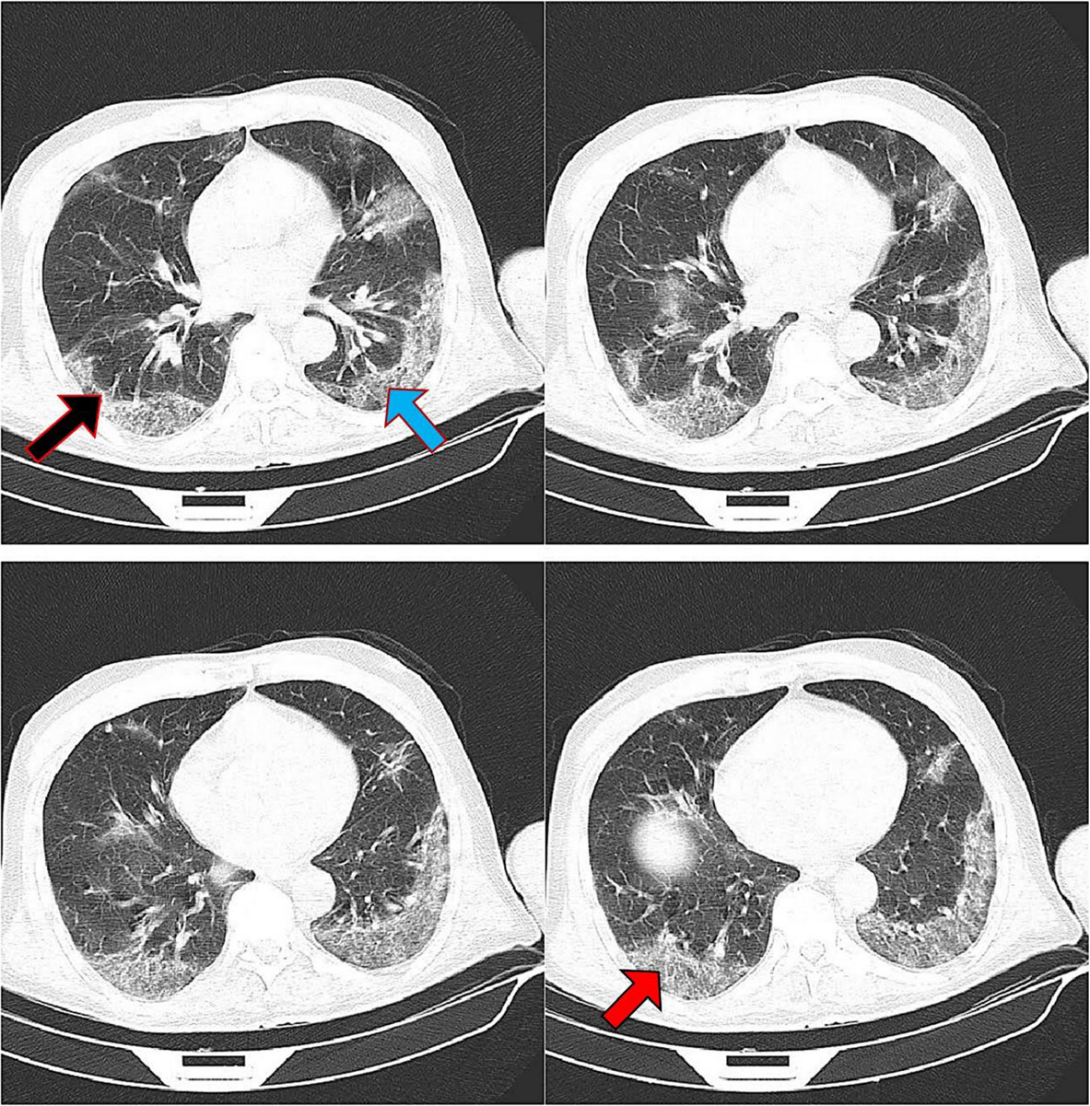
A 77-year-old male patient presented with fever, chills, and dry cough, who was admitted to the regular ward and was discharged on day 10 of admission. Peripheral multilobar ground glass opacities (red arrow), parenchymal bands (black arrow), and crazy paving (blue arrow) are observed in the CT scan. The total CT severity score was calculated as 15, and the disease severity was moderate

**Fig. 2:**
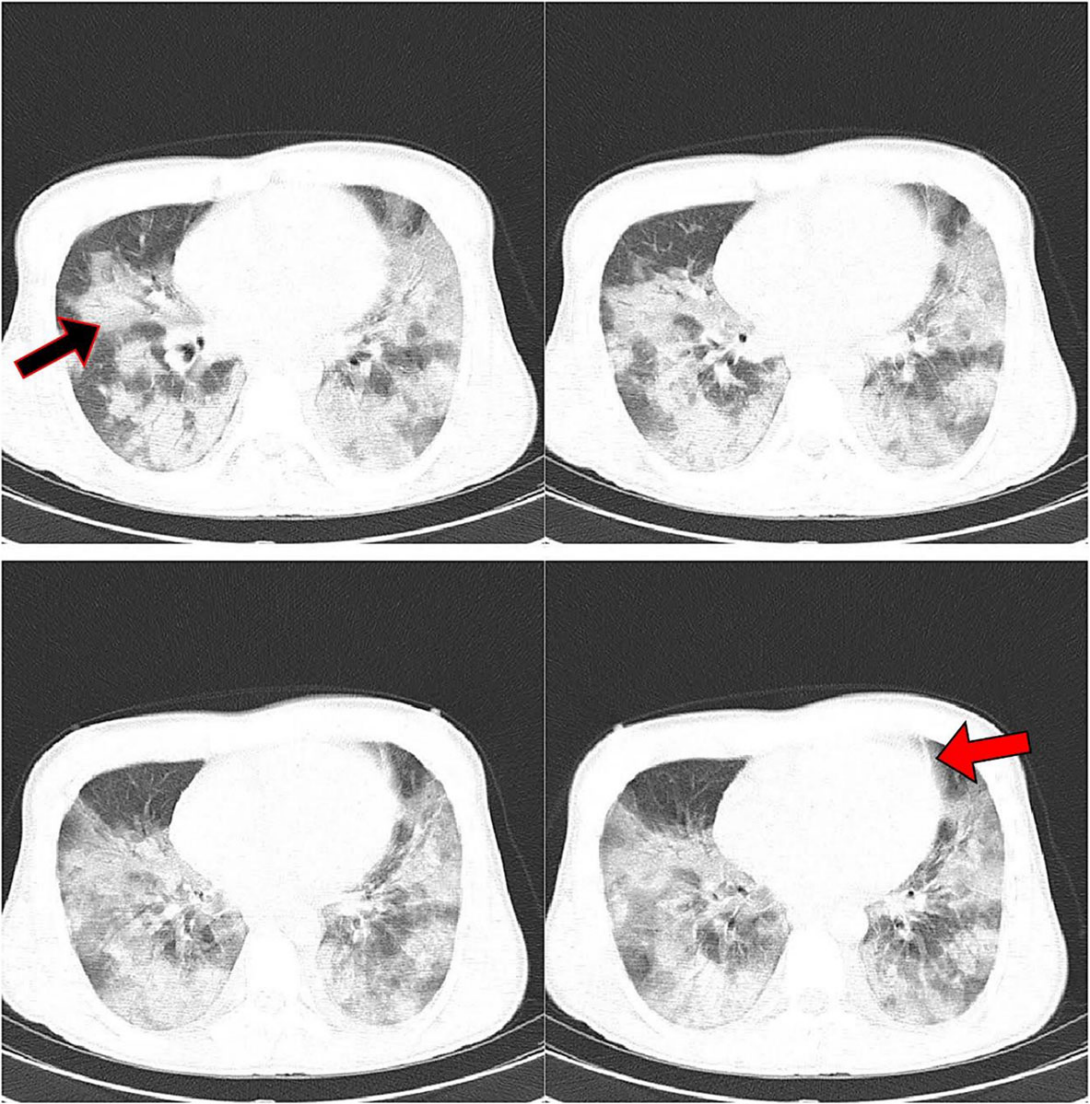
A 34-year-old male patient presented with fever, chills, myalgia, and dyspnea, who was admitted to ICU for 6 days and was discharged on day 21 of admission. Peripheral and peribronchovascular multilobar ground glass opacities (black arrow), and parenchymal bands (red arrow) are observed in the CT scan. The total CT severity score was calculated as 23, and the disease severity was severe

**Fig. 3:**
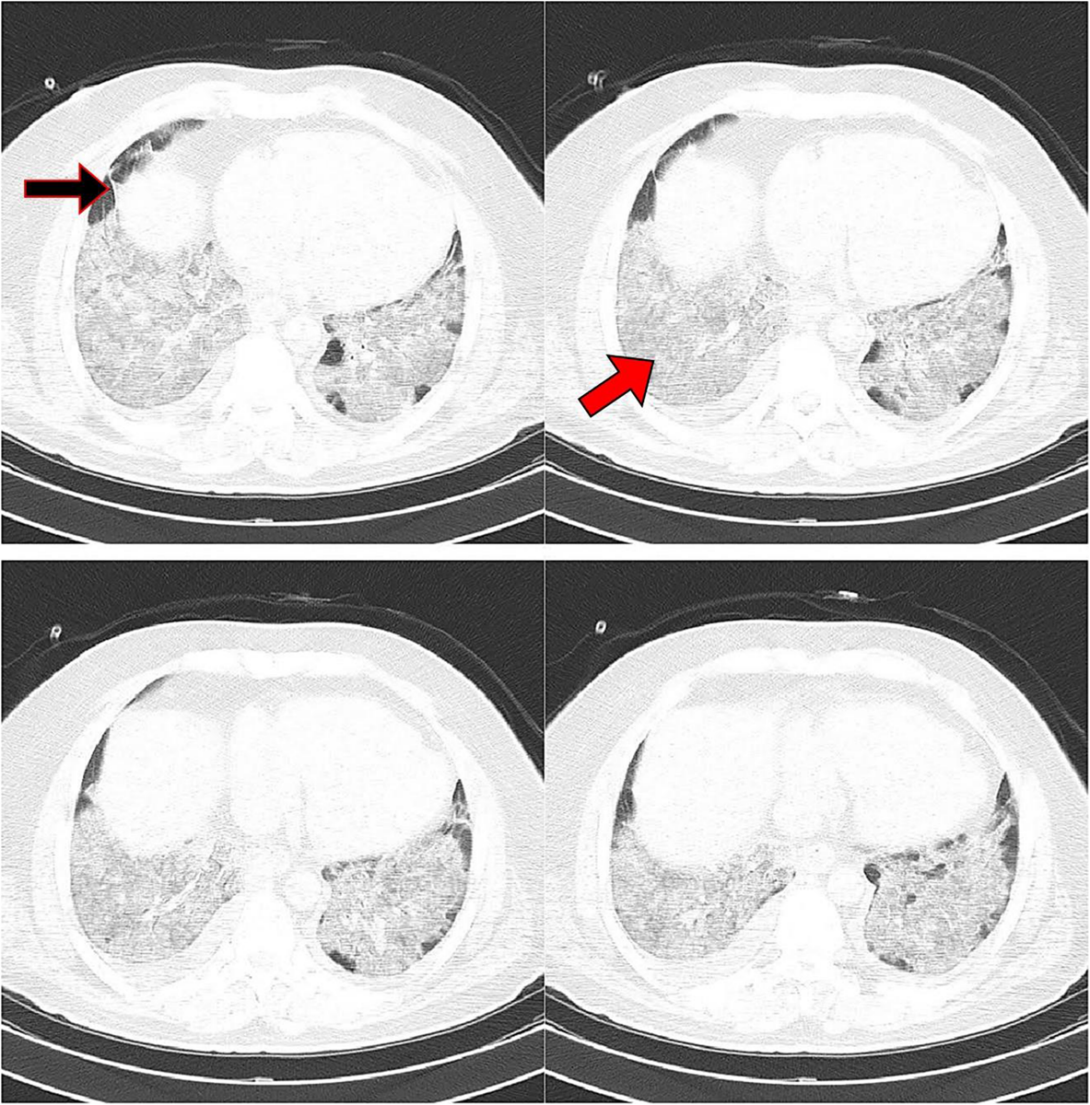
A 62-year-old male patient presented with fever, chills, myalgia, headache, and sputum discharge, who was admitted to ICU for 2 days and died. Extensive multilobar ground glass opacity and consolidation (red arrow), and parenchymal bands (black arrow) are observed in the CT scan, suggesting acute respiratory distress syndrome. The total CT severity score was calculated as 25, and the disease severity was critical

Of 523 patients with COVID-19, 317 (60.6%) were males and others were females. The mean age was 56.55 ± 14.75 years old. In Table 3, the characteristics of the RT-PCR-positive patients are represented according to ICU admission, mortality, and disease severity. The most frequent abnormal imaging findings observed in COVID-19 patients were ground glass (94.1%) and consolidation (91.0%). Table 4 exhibits the distribution of different CT scan findings by outcomes of COVID-19 patients. In this regard, ground glass opacity, consolidation, bilateral involvement, and peribronchovascular distribution were associated with ICU admission and disease severity. Also, consolidation and peribronchovascular distribution were significantly higher in non-survivors compared with survivors. The median number of zones involved and CSS were significantly higher in patients with the unfavorable outcomes than those without. Figures 1, 2, and 3 depict the CT scans of the patients with moderate, severe, and critical COVID-19 pneumonia, respectively.

**Table 3:** Distribution of baseline information by adverse clinical outcomes of COVID-19

Variables	ICU admission		***P*** value	Death		***P*** value	Disease severity^**a**^			***P*** value
	Yes	No		Yes	No		Moderate	Severe	Critical	
**Age (years), mean ± SD**	56.64 ± 13.66	56.51 ± 15.24	0.923	62.38 ± 13.04	54.79 ± 14.80	< 0.001	57.20 ± 15.46	54.39 ± 14.77	57.96 ± 13.07	0.084
**Sex, n (%)**										
**Males**	108 (66.3)	209 (58.1)	0.075	78 (64.5)	239 (59.5)	0.323	141 (58.3)	92 (59.4)	84 (66.7)	0.273
**Females**	55 (33.7)	151 (41.9)		43 (35.5)	163 (40.5)		101 (41.7)	63 (40.6)	42 (33.3)	
**CVDs, n (%)**										
**Yes**	79 (48.5)	140 (38.9)	0.040	58 (47.9)	161 (40.0)	0.123	98 (40.5)	55 (35.5)	66 (52.4)	0.014
**No**	84 (51.5)	220 (61.1)		63 (52.1)	241 (60.0)		144 (59.5)	100 (64.5)	60 (47.6)	
**Asthma/COPD, n (%)**										
**Yes**	27 (16.6)	25 (6.9)	0.001	16 (13.2)	36 (9.0)	0.169	18 (7.4)	9 (5.8)	25 (19.8)	< 0.001
**No**	136 (83.4)	335 (93.1)		105 (86.8)	366 (91.0)		224 (92.6)	146 (94.2)	101 (80.2)	
**Diabetes, n (%)**										
**Yes**	52 (31.9)	78 (21.7)	0.012	35 (28.9)	95 (23.6)	0.237	34 (14.0)	52 (33.5)	44 (34.9)	< 0.001
**No**	111 (68.1)	282 (78.3)		86 (71.1)	307 (76.4)		208 (86.0)	103 (66.5)	82 (65.1)	

*CVDs* cardiovascular diseases, *COPD* chronic obstructive pulmonary disease

^a^Based on the World Health Organization criteria

**Table 4:** Distribution of imaging findings by adverse clinical outcomes of COVID-19

Imaging findings	ICU admission		***P*** value	Death		***P*** value	Disease severity^**a**^			***P*** value
	Yes	No		Yes	No		Moderate	Severe	Critical	
**Ground glass, n (%)**										
Yes	159 (97.5)	333 (92.5)	0.024	114 (94.2)	378 (94.0)	0.940	219 (90.5)	150 (96.8)	123 (97.6)	0.005
No	4 (2.5)	27 (7.5)		7 (5.8)	24 (6.0)		23 (9.5)	5 (3.2)	3 (2.4)	
**Consolidation, n (%)**										
Yes	157 (96.3)	319 (88.6)	0.004	116 (95.9)	360 (89.6)	0.033	204 (84.3)	151 (97.4)	121 (96.0)	< 0.001
No	6 (3.7)	41 (11.4)		5 (4.1)	42 (10.4)		38 (15.7)	4 (2.6)	5 (10.6)	
**Reticular pattern, n (%)**										
Yes	49 (30.1)	80 (22.2)	0.54	32 (26.4)	97 (24.1)	0.604	48 (19.8)	45 (29.0)	36 (28.6)	0.059
No	114 (69.9)	280 (77.8)		89 (73.6)	305 (75.9)		194 (80.2)	110 (71.0)	90 (71.4)	
**Bilateral lung involvement, n (%)**										
Yes	162 (99.4)	335 (93.1)	0.002	119 (98.3)	378 (94.0)	0.055	226 (93.4)	145 (93.5)	126 (100)	0.013
No	1 (0.6)	25 (6.9)		2 (1.7)	24 (6.0)		16 (6.6)	10 (6.5)	0 (0)	
**Crazy paving, n (%)**										
Yes	40 (24.5)	71 (19.7)	0.212	30 (24.8)	81 (20.1)	0.273	44 (18.2)	40 (25.8)	27 (21.4)	0.193
No	123 (75.5)	289 (80.3)		91 (75.2)	321 (79.9)		198 (81.8)	115 (74.2)	99 (78.6)	
**Pleural effusion, n (%)**										
Yes	8 (4.9)	34 (9.4)	0.077	5 (4.1)	37 (9.2)	0.072	20 (8.3)	17 (11.0)	5 (4.0)	0.098
No	155 (95.1)	326 (90.6)		116 (95.9)	365 (90.8)		222 (91.7)	138 (89.0)	121 (96.0)	
**Lesions distribution, n (%)**										
Peribronchovascular										
Yes	78 (47.9)	97 (26.9)	< 0.001	58 (47.9)	117 (29.1)	< 0.001	54 (22.3)	58 (37.4)	63 (50.0)	< 0.001
No	85 (52.1)	263 (73.1)		63 (52.1)	285 (70.9)		188 (77.7)	97 (62.6)	63 (50.0)	
Peripheral										
Yes	157 (96.3)	347 (96.4)	0.968	119 (98.3)	385 (95.8)	0.184	234 (96.7)	148 (95.5)	122 (96.8)	0.781
No	6 (3.7)	13 (3.6)		2 (1.7)	17 (4.2)		8 (3.3)	7 (4.5)	4 (3.2)	
**Number of zones involved, median (IQR)**	5 (4-5)	4 (3–5)	< 0.001	5 (4–5)	4 (4–5)	0.002	4 (3–5)	5 (4-5)	5 (4–5)	< 0.001
**CSS (continuous), median (IQR)**	17 (11-22)	22 (18–24)	< 0.001	18 (12–22)	22 (17–24)	< 0.001	15 (10.75–20.25)	20 (15–23)	23 (18–25)	< 0.001
**CSS ≥ 19, n (%)**										
Yes	115 (70.6)	150 (41.7)	< 0.001	76 (62.8)	189 (47.0)	0.002	79 (32.6)	93 (60.0)	93 (73.8)	< 0.001
No	48 (29.4)	210 (58.3)		45 (37.2)	213 (53.0)		163 (67.4)	62 (40.0)	33 (26.2)	

*IQR* interquartile range, *CSS* CT severity score

^a^Based on the World Health Organization criteria

According to logistic regression model, peribronchovascular distribution of lesions, number of zones involved, and CSS were associated with increased risk of ICU admission (OR = 2.93, OR = 2.10, and OR = 1.14, respectively; AUC = 60.5%, AUC = 66.6%, and AUC = 71.6%, respectively), mortality (OR = 2.30, OR = 1.35, and OR = 1.08, respectively; AUC = 59.4%, AUC = 57.9%, and AUC = 64.4%, respectively), severe disease (OR=2.06, OR = 1.68, and OR = 1.10, respectively; AUC = 52.8%, AUC = 58.2%, and AUC = 54.5%, respectively), and critical disease (OR = 4.62, OR = 3.21, and OR = 1.23, respectively; AUC = 60.9%, AUC = 65.6%, and AUC = 74.6%, respectively) (Table 5). Also, patients who had consolidation were at a higher risk of severe disease compared with those who did not (OR = 4.94). The median number of CSS was 19 in COVID-19 patients and we used it as a threshold for the relevant analyses. According to the analyses, CSS ≥ 19 could be predictive for ICU admission (OR = 3.21, AUC = 64.4%), mortality (OR = 1.84, AUC = 57.9%), severe disease (OR = 3.18, AUC = 56.6%) and critical disease (OR = 6.25, AUC = 65.2%) (Table 5).

**Table 5:** Association between imaging findings and adverse clinical outcomes of COVID-19

Outcomes	Imaging findings	Adjusted^**a**^ OR (95% CI)	***P*** value
**ICU admission**	Ground glass	0.42 (0.09–1.99)	0.274
	Consolidation	2.23 (0.62–7.95)	0.218
	Bilateral lung involvement	3.66 (0.38–35.23)	0.262
	Peribronchovascular distribution	2.93 (1.92–4.47)	< 0.001
	Involved zones	2.10 (1.55–2.86)	< 0.001
	CSS (continuous)	1.14 (1.10–1.19)	< 0.001
	CSS ≥ 19	3.21 (2.15–4.81)	< 0.001
**Mortality**	Consolidation	1.72 (0.63–4.65)	0.288
	Peribronchovascular distribution	2.30 (1.50–3.53)	< 0.001
	Involved zones	1.35 (1.05–1.74)	0.020
	CSS (continuous)	1.08 (1.04–1.12)	< 0.001
	CSS ≥ 19	1.84 (1.21–2.81)	0.004
**Disease severity**^**b**^			
Severe	Ground glass	0.51 (0.13–2.02)	0.337
	Consolidation	4.94 (1.22–20.02)	0.025
	Peribronchovascular distribution	2.06 (1.26–3.35)	0.004
	Involved zones	1.68 (1.29–2.18)	0.001
	CSS (continuous)	1.10 (1.06–1.14)	< 0.001
	CSS ≥ 19	3.18 (2.05–4.93)	< 0.001
Critical	Ground glass	0.28 (0.04–1.89)	0.195
	Consolidation	3.06 (0.74–12.68)	0.123
	Peribronchovascular distribution	4.62 (2.64–8.10)	< 0.001
	Involved zones	3.21 (2.25–4.58)	< 0.001
	CSS (continuous)	1.23 (1.16–1.29)	< 0.001
	CSS ≥ 19	6.25 (3.74–10.41)	< 0.001

*OR* odds ratio, *CI* confidence interval

^a^Adjusted for patients’ comorbidities (cardiovascular diseases, asthma/chronic obstructive pulmonary disease, and diabetes) and imaging findings

^b^Based on the World Health Organization criteria. “Moderate” disease was reference

## Discussion

In the present study, we investigated the potential predictive ability of initial chest CT scan findings for COVID-19 status and adverse clinical outcomes. It was found that cases with ground glass opacity had a two-fold increased likelihood of COVID-19 infection compared with those without. This CT finding had also acceptable sensitivity and accuracy. In the study by Chen et al. [12], consolidation was reported to be predictive for COVID-19 infection, which was inconsistent with our results. A superiority of the present study to the mentioned survey is a larger sample size. Concerning the diagnostic performance of CT scan, a recent meta-analysis reported sensitivity and specificity of 87% and 46%, respectively. Despite a good diagnostic sensitivity, this meta-analysis recommended to use RT-PCR besides CT scan to reach the most accurate result [7].

We found that CSS could be predictive for COVID-19 infection, that is, a higher total CSS is directly correlated to increased odds of the disease. Similar to our results, the study by Al-Mosawe et al. [13] showed that the probability of RT-PCR positivity increases with increase in CT score. To the best of our knowledge, limited number of studies have explored the association between CSS and COVID-19 status. Our results propose that CSS can be used in combination with clinical examination for the initial management of the suspected COVID-19 cases while waiting for RT-PCR results. It would be useful for clinicians and radiologists to reach a consensus on a threshold for CSS to better identify the COVID-19 cases.

With respect to the adverse outcomes of COVID-19, we assessed whether the initial CT scan findings inform ICU admission, mortality, and disease severity. In this regard, we found that peribronchovascular distribution of lesions, number of lung zones involved, and total CSS were associated with increased risk of the unfavorable outcomes. Also, consolidation was demonstrated to predict severe COVID-19 disease. Lei et al. [14] showed that a higher CT score was associated with an increased odds of mortality, which was in agreement with our results. On the other hand, number of lung zones involved did not predict mortality, which was not consistent with our findings. In the study by Liu et al. [8], which used the same criteria as the present study used for the disease severity (WHO), number of lung lobes involved and total CT score were directly correlated to disease severity. In other study, it was stated that the odds of adverse outcome (need for mechanical ventilation or mortality) is four times higher in patients with more than four lung zones involved than in those without [15]. Auger et al. [16] reported that ground glass, crazy paving, and consolidation did not have a significant association either with invasive endotracheal ventilation or mortality. On the other hand, number of lung zones involved was associated with invasive endotracheal ventilation, but not with death.

As observed, there are conflicting results between studies on the predictive ability of CT scan findings for clinical outcomes of COVID-19. However, CSS is apparently able to predict the prognosis of the patients with COVID-19. It is suggested to approve a threshold for CSS to discriminate high-risk from low-risk patients. In the present study, we considered a threshold for the unfavorable outcomes and witnessed relatively strong associations between these two study outcomes and CSS classification.

A limitation of this study was that we only used the initial CT scan without repetition. Thus, it is suggested to perform longitudinal studies to prospectively re-evaluate the patients with more details for testing generalizability. Also, considering that a single radiologist reviewed the CT scans, it is suggested that at least two senior radiologists contribute in reviewing the CT findings in the further studies.

## Conclusion

According to the results, initial CT severity scores could predict positive COVID-19 status, ICU admission, mortality, and disease severity. Moreover, peribronchovascular distribution of the lesions and number of lung zones involved predicted adverse outcomes of COVID-19 infection. Having consolidation was also directly associated with severe disease. Initial CT scores potentially have diagnostic and prognostic values that can facilitate triage of the cases suspected of COVID-19 and/or management of the patients diagnosed with the infection for clinicians.

## Data Availability

The datasets during and/or analyzed during the current study are available from the corresponding author on a reasonable request.

## Abbreviations

COVID-19: Coronavirus disease 2019
SARS-CoV-2: Severe acute respiratory syndrome coronavirus 2
RT-PCR: Real-time polymerase chain reaction
CT: Computed tomography
CVDs: Cardiovascular diseases
COPD: Chronic obstructive pulmonary disease
WHO: World Health Organization
CSS: CT severity score
OR: Odds ratio

## References

[1] Lai C-C, Shih T-P, Ko W-C, Tang H-J, Hsueh P-R (2020). Severe acute respiratory syndrome coronavirus 2 (SARS-CoV-2) and coronavirus disease-2019 (COVID-19): The epidemic and the challenges. Int J Antimicrob Agents.

[2] Johns Hopkins University. COVID-19 Dashboard by the Center for Systems Science and Engineering (CSSE) at Johns Hopkins University. Global Map; 2021. Available at: https://coronavirus.jhu.edu/map.html. (Accessed 25 Apr 2021).

[3] Wu J, Liu J, Li S, Peng Z, Xiao Z, Wang X, Yan R, Luo J (2020). Detection and analysis of nucleic acid in various biological samples of COVID-19 patients. Travel Med Infect Dis.

[4] Tahamtan A, Ardebili A (2020). Real-time RT-PCR in COVID-19 detection: issues affecting the results. Expert Rev Mol Diagn.

[5] Li K, Wu J, Wu F, Guo D, Chen L, Fang Z, Li C (2020). The clinical and chest CT features associated with severe and critical COVID-19 pneumonia. Invest Radiol.

[6] Simpson S, Kay FU, Abbara S, Bhalla S, Chung JH, Chung M, Henry TS, Kanne JP, Kligerman S, Ko JP, Litt H (2020). Radiological society of north America expert consensus document on reporting chest CT findings related to COVID-19: endorsed by the society of thoracic Radiology, the American college of Radiology, and RSNA. Radiol: Cardiothor Imaging.

[7] Khatami F, Saatchi M, Zadeh SST, Aghamir ZS, Shabestari AN, Reis LO, Aghamir SMK (2020). A meta-analysis of accuracy and sensitivity of chest CT and RT-PCR in COVID-19 diagnosis. Sci Rep.

[8] Liu Z, Jin C, Wu CC, Liang T, Zhao H, Wang Y, Wang Z, Li F, Zhou J, Cai S, Zeng L, Yang J (2020). Association between initial chest CT or clinical features and clinical course in patients with coronavirus disease 2019 pneumonia. Korean J Radiol.

[9] Jiang M, Chen P, Li T, Tang Y, Chen X, Chen X, Ruan X (2021). Chest CT imaging features and clinical outcome of coronavirus disease 2019 (COVID-19): A single-center case study in Ningbo, China. Clin Imaging.

[10] World Health Organization. COVID-19 Clinical management: living guidance. Available at: https://www.who.int/publications/i/item/WHO-2019-nCoV-clinical-2021-1. (Accessed 25 Jan 2021).

[11] Pan F, Ye T, Sun P, Gui S, Liang B, Li L, Zheng D, Wang J, Hesketh RL, Yang L, Zheng C (2020). Time course of lung changes at chest CT during recovery from coronavirus disease 2019 (COVID-19). Radiology.

[12] Chen D, Jiang X, Hong Y, Wen Z, Wei S, Peng G, Wei X (2021). Can chest CT features distinguish patients with negative from those with positive initial RT-PCR results for coronavirus disease (COVID-19)?. Am J Roentgenol.

[13] Al-Mosawe AM, Mohammed Abdulwahid H, Fayadh NAH (2021) Spectrum of CT appearance and CT severity index of COVID-19 pulmonary infection in correlation with age, sex, and PCR test: an Iraqi experience. Egypt J Radiol Nucl Med 52(1). 10.1186/s43055-021-00422-3

[14] Lei Q, Li G, Ma X, Tian J, Fan Wu Y, Chen H (2021). Correlation between CT findings and outcomes in 46 patients with coronavirus disease 2019. Sci Rep.

[15] Liu S, Nie C, Xu Q, Xie H, Wang M, Yu C, Hou X (2021). Prognostic value of initial chest CT findings for clinical outcomes in patients with COVID-19. Int J Med Sci.

[16] Auger R, Dujardin P-A, Bleuzen A, Buraschi J, Mandine N, Marchand-Adam S (2021). Chest computed tomography signs associated with pejorative evolution in COVID-19 patients. Polish Journal of Radiology.

